# Intrapartum ultrasound measurement of the lower uterine segment thickness in parturients with previous scar in labor: a cross-sectional study

**DOI:** 10.1186/s12884-022-04747-3

**Published:** 2022-05-14

**Authors:** Shahla K. Alalaf, Tarek Mohamed M. Mansour, Sileem Ahmad Sileem, Nazar P. Shabila

**Affiliations:** 1 Department of Obstetrics and Gynecology, College of Medicine, Hawler Medical University, Kurdistan Region, Erbil city, Iraq; 2 Department of Radiology, Faculty of Medicine, Al-Azhar University, Assuit, Egypt; 3 Department of Obstetrics and Gynecology, Faculty of Medicine, Al-Azhar University, Assuit, Egypt; 4 Department of Community Medicine, College of Medicine, Hawler Medical University, Kurdistan Region, Erbil City, Iraq

**Keywords:** Cesarean section, Myometrium thickness, Lower uterine segment, Uterine rupture

## Abstract

**Background:**

There is a lack of reliable methods to estimate the risk of uterine rupture or dehiscence during a trial of labor in women with previous cesarean sections. This study aimed to assess the lower uterine segment and myometrial thickness by ultrasonography in women with previous cesarean sections during labor and assess their association with the uterine defect.

**Methods:**

A cross-sectional study was conducted on 161 women in the active phase of labor having one previous cesarean section. The study was conducted et al.-Azhar University Hospital, Assiut City, Egypt, from March 2018 to March 2019. Ultrasound measurements of lower uterine segment thickness and myometrial thickness were conducted by vaginal and abdominal ultrasound by two observers. The correlation of both thicknesses with the uterine defect was analyzed.

**Results:**

Uterine defects were reported in 42 women (25.9%), uterine rupture in four women (2.5%), and dehiscence in 38 women (23.5%). The uterine defects were not associated with maternal factors (maternal age, gestational age at labor, body mass index, birth weight, interpregnancy, and inter-delivery interval). Receiver operating curve analysis demonstrated that lower uterine segment thickness was linked with uterine defect, with an area under the curve of 60% (95% CI, 51–70%, *P* = 0.044). Myometrial thickness was also linked to the uterine defect, with an area under the curve of 61% (95% CI, 52–71%, *P* = 0.025). Full lower uterine segment thickness of 2.3 mm and myometrial thickness of 1.9 mm were the cutoff value with the best combination of sensitivity and specificity for the uterine defect. Lower uterine segment thickness (OR = 0.49, 95%CI 0.24–0.96) and myometrial thickness (OR = 0.44, 95%CI 0.20–0.94) were significantly associated with the uterine defect. Lower uterine segment thickness (OR = 0.41, 95%CI 0.22–0.76) and myometrial thickness (OR = 0.33, 95%CI 0.16–0.66) were also significantly associated with cesarean section delivery.

**Conclusion:**

A lower uterine segment thickness of 2.3 mm and myometrial thickness of 1.9 mm during the first stage of labor are associated with a high risk of uterine defects during a labor trial. These measurements during labor can have a practical application in deciding the mode of delivery in women with previous cesarean sections and might reduce uterine rupture.

## Background

The cesarean section rate has increased continuously almost all over the world [1]. Vaginal birth after cesarean section is a dominant upcoming mode of delivery compared to elective repeat cesarean delivery as it is less costly and more effective [2]. It is well known that failed vaginal birth after a cesarean section has higher risks of uterine disruption and infectious morbidity than patients who have a successful vaginal birth after cesarean section or elective repeat cesarean delivery [3]. The integrity and thickness of the previous cesarean section scar of the lower uterine segment (LUS) assessed by ultrasound is a popular strategy that has been proposed to estimate the likelihood of uterine rupture or dehiscence that might occur spontaneously or during labor trial; this can be done alone or in combination with clinical factors [4].

Antenatal care is recognized as an important opportunity for screening and early identification of pregnancy complications and planning for the mode of delivery [5]. A significant proportion of women from developing countries do not start antenatal care according to the World Health Organization recommendations [6, 7]; they attend less and usually have their first antenatal care visit late in pregnancy [8]. Besides that, access to obstetrical emergency care in developing countries and the presence of skilled personnel and equipment is often limited. Thus, women with a previous scar in low-income countries may present for the first time to the hospital in labor. This makes deciding on the delivery plan's mode very critical [9].

There is minimal evidence to predict uterine rupture or dehiscence using ultrasound or clinical variables. Furthermore, there is a lack of reliable methods to estimate the risk of uterine rupture or dehiscence during a trial of labor in women with previous cesarean sections [4, 10]. The available published articles had used ultrasound to estimate the full LUS thickness [11–13], or the myometrial thickness (MT) [14, 15], or both of them [16] to predict uterine dehiscence. All these were conducted in the late stage of pregnancy, and they correlate the outcome regarding uterine dehiscence either after successful vaginal birth, after cesarean section, or during elective repeat cesarean delivery.

No studies have been conducted during labor to assess the LUS and MT by ultrasonography to our best knowledge. This study aimed to assess the LUS and MT in women with a previous cesarean section presented in active labor in a very busy hospital in Egypt and assess their association with uterine rupture and dehiscence.

## Methods

This cross-sectional study was conducted on 162 women in the active phase of labor and having one previous cesarean section. The study was conducted et al.-Azhar University Hospital, Assiut City, Egypt, from March 2018 to March 2019, including data collection and analysis, follow-up of women, and manuscript writing. Approval to conduct this study was obtained from the Al-Azhar University Ethical Committee on 10th March 2018 (No.3.5.2018). Written informed consent was obtained from all the participants.

Al-Azhar University Hospital provides various obstetrics services such as managing high-risk pregnancies and deliveries, including cesarean sections and medical termination of pregnancy. The hospital serves the entire population of the Assiut government [17]. According to the Directorate of Health in Assiut city, 20,167 deliveries occurred et al.-Azhar University Hospital in 2018. Around half of all births occur at home in Egypt [18]. The majority of pregnant women attending Assiut University Hospital are from rural areas, with inadequate antenatal visits and low socioeconomic status [19].

### Inclusion and exclusion criteria

Women between 33 weeks 7 days and 41 weeks 6 days of gestation with a singleton pregnancy in cephalic presentation, having one previous low transverse cesarean section delivery, being in the active stage of labor (cervix dilated 4 cm and up to 8 cm), and accept to participate were included in the study. Women having twin pregnancies, less than 34 weeks gestation, previous two cesarean sections, intrauterine fetal demise, 8 cm to fully dilated cervix, and refused to participate in the trial were excluded.

### Participants

Data about the maternal characteristics were collected, including age, BMI (kg/ m2), parity, variables related to the previous cesarean delivery such as interpregnancy and inter delivery intervals, any successful trial of labor, and medical history. Variables related to current delivery were also collected, such as gestational age, the progress of labor, and indications for cesarean section. The mode of delivery was recorded, being vaginal birth after cesarean section or emergency cesarean section. The obstetrician on call examined participants; the trial of labor was decided by him, and the indications for the emergency cesarean section. The obstetrician was blinded to the study's objectives and sonographic findings regarding LUS and MT.

### Sample size estimation

The sample size was calculated using the Epi-info, assuming that the prevalence of uterine rupture among pregnant women with LUS thickness of less than 3 mm is 18.6%, according to a pilot study on 20 delivering women. We found that a sample size of 162 pregnant women was sufficient to achieve a 95% confidence interval for the prevalence with ± 6% precision.

### Procedure

The obstetrician on call requested the ultrasound examination. The ultrasound procedure was conducted by two experts in ultrasound with experience in the estimation of the thickness of the lower segment. The sonographic examination was performed using (GE voluson p6 ultrasound machine, Korean 2007) with 3.75 MHz curvilinear transducer when abdominal ultrasound was used and a transvaginal probe 7.5 MHzon when the vaginal estimation of the thickness was used. The LUS and MT were measured trans abdominally for the full LUS thickness with the bladder being full. Then, a transvaginal ultrasound was conducted to confirm the results with the bladder being empty. Normal ultrasound features of the LUS are a two-layered structure that consists of a superficial echogenic layer, the outer myometrium, and a deep, less echogenic layer, which involves both the inner myometrium and the decasualized endometrium.

A longitudinal, transverse scan was conducted to determine any dehiscence of the lower segment. Then sagittal sections were measured (three measures on average) to find the thinnest zone of the lower segment [16].

**Fig. 1:**
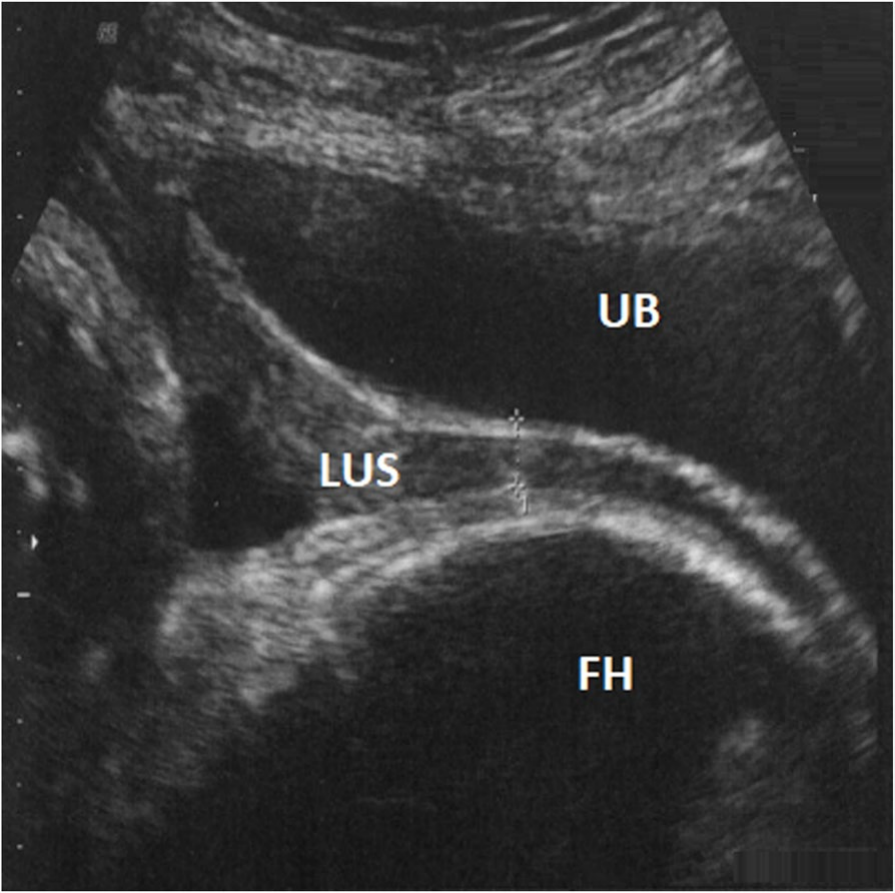
Trans vaginal ultrasound during first stage of labor with empty bladder, measuring the Lower Uterine Segment (LUS) and the LUS with posterior UB wall, which shows a normal LUS thickness (2.5 mm). M: myometrium, F: fetus head

**Fig. 2:**
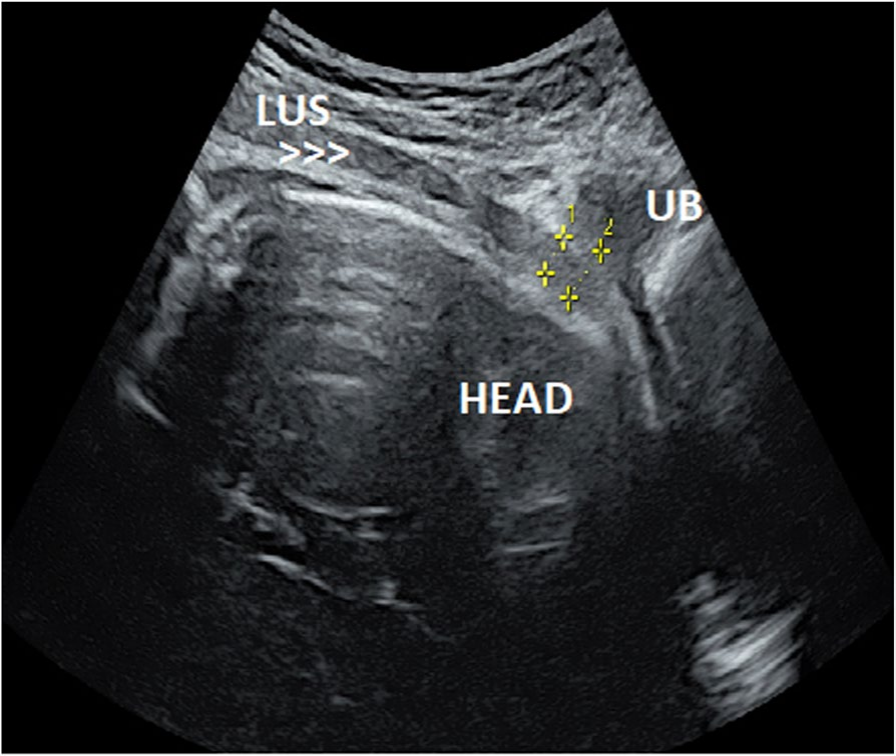
Trans abdominal ultrasound during first stage of labor with partial empty bladder, measuring the Lower Uterine Segment (LUS) with posterior UB wall, which shows a decreased thickness of the LUS (1.5 mm). M: myometrium, F: fetus head

Full LUS thickness was defined as the distance between the amniotic cavity and the bladder wall; this was measured by placing one caliper at the interface between urine and bladder wall and the other at the interface between decidual endometrium and amniotic fluid. MT was defined as the least thickness covering the amniotic cavity at the level of the uterine scar, where only the myometrium was measured [20, 21]. Figures 1 and 2 illustrate transvaginal and transabdominal ultrasound during the first stage of labor that shows the measurement of LUS and MT. Figure 1 shows an example of transvaginal ultrasound of normal LUS thickness (2.5 mm), while Fig. 2 shows an example of transabdominal ultrasound of decreased thickness of LUS (1.5 mm). The measurements were conducted during uterine retraction as it was too difficult to accomplish it during uterine contraction due to the pain and stress of labor.

The reproducibility of ultrasound measurements of the thickness of the LUS using vaginal and abdominal ultrasound and of the two observers was not conducted as previously published research has shown that the intra- and inter-observer differences were 1 mm or less [22, 23].

### Follow up of participants

Follow up of women during labor was performed with the obstetrician on call and professional midwives according to hospital regulations. All the labor and delivery outcomes were reviewed.

The assessments of the uterine scar by the obstetrician at delivery were compared with the sonographic results. When the emergency cesarean section was conducted for fetal and or maternal indication, lower segment dehiscence or rupture was confirmed by naked eye appearance during operation. The assessment was straightforward as the uterine rupture was defined as a complete separation of the uterine scar with direct communication with peritoneal cavities. Uterine dehiscence was diagnosed by the naked eye appearance of a subperitoneal separation of the uterine scar and seeing the chorioamniotic membrane through the peritoneum of the LUS [24].

Manual uterine exploration after vaginal birth after the cesarean section was conducted only on symptomatic women suspicious of uterine rupture (excessive vaginal bleeding and signs of hypovolemia). Uterine manipulation revealed one rupture uterus and four just thinning of the lower segment (uterine dehiscence).

### Statistical analysis

We used the statistical package for the social sciences for data management and analysis. Each maternal factor, such as age, gestational age, birth weight, and inter pregnancy interval, were divided into different groups for data presentation and analysis purposes. Determination of the cutoff points for such grouping has been based on the documented association of some groups or levels of these factors with adverse maternal and fetal outcomes. For example, the recommended inter pregnancy interval is 18–24 months, and intervals shorter than 18 months appear to be associated with increased adverse outcomes, including preterm delivery, small-for-gestational-age birth, and infant mortality [25, 26]. Therefore, the inter pregnancy interval was divided into ≤ 24 months, which may be associated with predicting uterine defect in women with previous cesarean section and in labor, and > 24 months. The same principle was applied to the other variables.

The student's t-test was used to compare two means, and the Chi-square test was used to compare proportions. Receiver operating curve analyses were used to determine optimal cutoff values for sensitivity and specificity [16]. Multivariate analysis was carried out based on binary logistic regression to adjust for and examine the independent effects of possible covariates. Baseline variables were considered for inclusion in the multivariate model. They were based on a significant univariate test based on the Wald test from the logistic regression with a P value cutoff point of 0.25 considering the threshold for including variables in the multivariate model [27]. All of the non-significant and non-confounder covariates were removed from the model. The level of statistical significance was set at P value ≤ 0.05.

## Results

Uterine rupture was reported in four women (2.5%), while dehiscence was reported in 38 women (23.5%). Thus, uterine defect (rupture and dehiscence) was reported in 42 women (25.9%). The diagnosis of uterine rupture or dehiscence was made mainly during the cesarean Sect. (88.1%). Most women had a gestational age of 37–38 weeks (37.7%). The mean inter-pregnancy interval was 40.6 ± 13.7 months (14–84 months). The mean inter-delivery interval was 49.2 ± 13.8 months. The mean LUS thickness was 2.28 ± 0.52 mm (range 1.5 – 3.0), and the mean MT was 1.77 ± 0.46 mm (range 1.10 – 2.60). 57.4% had a vaginal delivery, and 42.6% had a cesarean section. The vaginal birth after cesarean section success rate was 54.9%. The main indications for the cesarean section included the impending rupture of the uterus (42.6%).

The mean ± SD of LUS thickness was significantly lower in uterine defect cases than the normal cases (2.23 ± 0.520 vs. 2.43 ± 0.503, *P* = 0.036). The mean ± SD of MT thickness was significantly lower in uterine defect cases than the normal cases (1.73 ± 0.457 vs. 1.90 ± 0.441, *P* = 0.033). All the other factors were not significantly associated with uterine defects, as shown in Table 1.

**Table 1:** Association between uterine defect and the clinical characteristics of the participants

Variable	Uterine defect				*P* value
	**Normal**		**Defect**		
	**No**	**%**	**No**	**%**	
**LUS thickness mm (Mean and SD)**	2.43	0.503	2.23	0.520	0.036
**Myometrial thickness mm (Mean and SD)**	1.90	0.441	1.73	0.457	0.033
**Age group (years)**					
19–25	26	76.5%	8	23.5%	0.905
26–30	55	74.3%	19	25.7%	
≥ 31	39	72.2%	15	27.8%	
**Gestation age (weeks)**					
34–36	39	84.8%	7	15.2%	0.123
37–38	44	72.1%	17	27.9%	
39–41	37	67.3%	18	32.7%	
**Birth weight**					
Low (1.2–2.4 kg)	18	85.7%	3	14.3%	0.192
Normal (2.5–3.9 kg)	102	72.3%	39	27.7%	
**BMI (kg/m**^**2**^**)**					
18.5–24.9 (Normal)	56	77.8%	16	22.2%	0.524
25–29.9 (Overweight)	48	69.6%	21	30.4%	
≥ 30 (Obese)	16	76.2%	5	23.8%	
**Inter pregnancy interval**					
≤ 24 months	15	71.4%	6	28.6%	0.767
> 24 months	105	74.5%	36	25.5%	
**Inter delivery interval**					
≤ 36 months	29	70.7%	12	29.3%	0.572
> 36 months	91	75.2%	30	24.8%	

*LUS* Lower uterine segment, *BMI* Body mass index

**Fig. 3:**
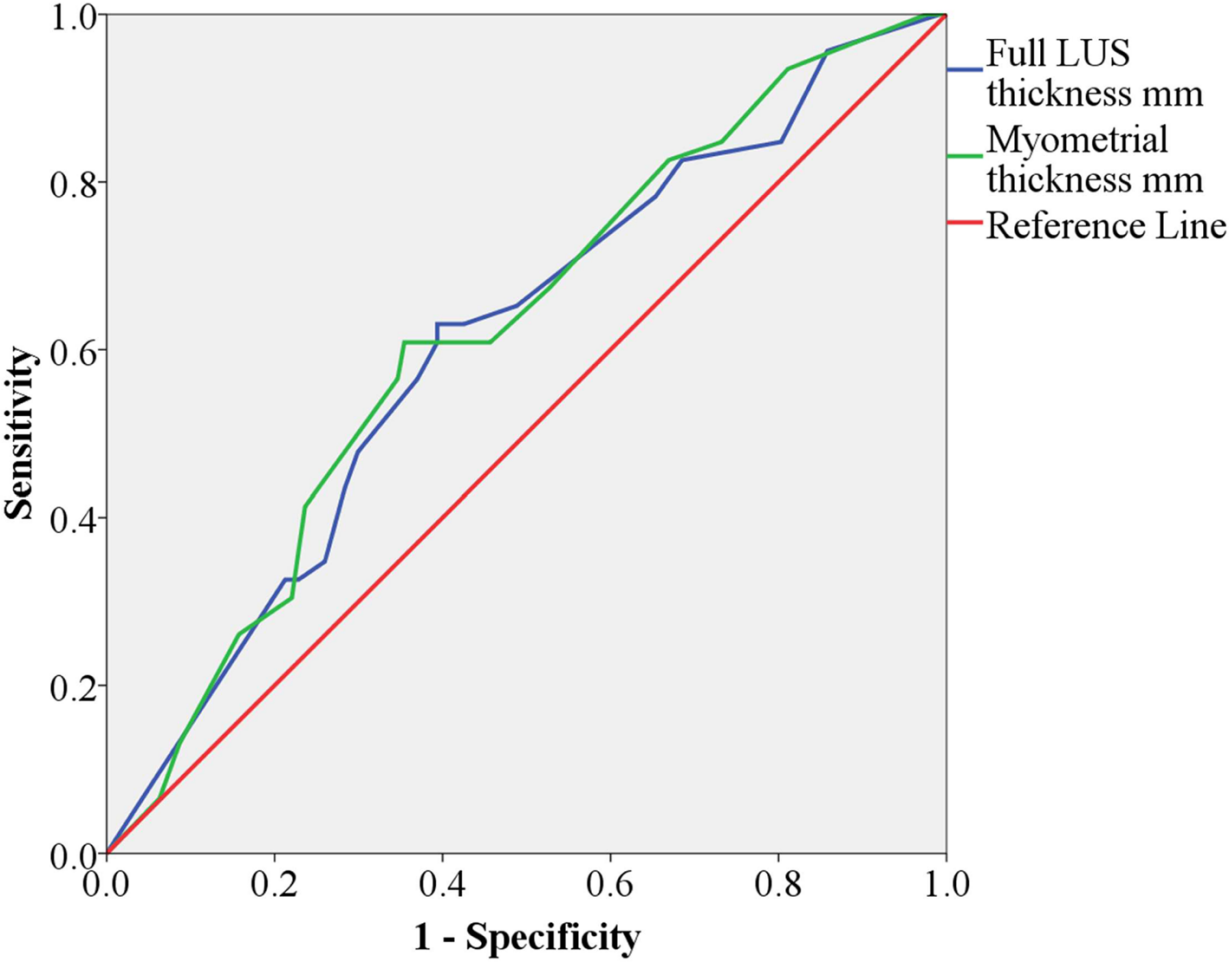
Receiver operative curve (ROC) comparing the sensitivity and specificity of lower uterine segment (LUS) thickness and myometrial thickness (MT) with the uterine defect

Receiver operating curve analysis demonstrated that LUS thickness was linked with uterine defect, with an area under the curve of 60% (95% confidence interval [CI], 51–70%, *P* = 0.044). MT was also linked to the uterine defect, with an area under the curve of 61% (95% CI, 52–71%, *P* = 0.025), as shown in Fig. 3. The cutoff values of full LUS and MT were determined by selecting the values that produced the highest sensitivity plus specificity combination value. Full LUS thickness of 2.3 mm was the cutoff value with the best combination of sensitivity and specificity (62% and 62%, respectively) for the uterine defect. MT of 1.9 mm was the cutoff value with the best combination of sensitivity and specificity (60% and 65%, respectively) for the uterine defect.

Multivariate analysis showed that the indications of cesarean section were significantly associated with uterine defect (OR = 33, 95%CI 3.36–323.8 for second stage difficulty and OR = 5, 95%CI 1.45–17.27 for impending uterine rupture compared with the first stage difficulty). Increasing LUS thickness was significantly associated with less uterine defect (OR = 0.49, 95%CI 0.24–0.96) and cesarean section (OR = 0.41, 95%CI 0.22–0.76). Increasing MT thickness was significantly associated with fewer uterine defects (OR = 0.44, 95%CI 0.20–0.94) and cesarean section (OR = 0.33, 95%CI 0.16–0.66), as shown in Table 2.

**Table 2:** Logistic regression analysis for factors associated with uterine defect and the need for cesarean section

**Uterine defect**					
**Factor**	**B**	***P*** **value**	**Odds ratio**	**95% CI**	
				**Lower**	**Upper**
Parity	-0.029	0.848	0.97	0.72	1.31
First stage difficulty			Ref		
Second stage difficulty	3.50	0.003	33.0	3.36	323.81
Impending rupture	1.61	0.011	5.0	1.45	17.27
LUS thickness	-0.724	0.038	0.49	0.24	0.96
Myometrial thickness	-0.833	0.035	0.44	0.20	0.94
**Cesarean section**					
**Factor**	**B**	***P*** **value**	**Odds ratio**	**95% CI**	
				**Lower**	**Upper**
Parity	0.020	0.885	1.02	0.78	1.33
LUS thickness	-0.894	0.005	0.41	0.22	0.76
Myometrial thickness	-1.125	0.002	0.33	0.16	0.66

*LUS* Lower uterine segment

## Discussion

The primary finding of this study is that LUS thickness and MT measured by ultrasound during the active stage of labor in women with one previous transverse lower segment cesarean section may help in predicting complete uterine rupture with a LUS thickness of 2.3 mm and less and MT of 1.9 mm and less.

The clinical usefulness of the results might be important because they may help to identify a subgroup of women at high risk of uterine rupture, mostly being unplanned to have a vaginal birth after a previous cesarean section. As we face this situation in maternity hospitals in developing countries, such results can influence decision-making between vaginal birth after cesarean section versus emergency cesarean section. Moreover, research has shown that ultrasound measurement of LUS thickness at late pregnancy did not result in a statistically significant lower frequency of maternal and perinatal adverse outcomes than standard management [13].

The association between sonographic LUS thickness during late pregnancy and uterine rupture has been looked for in many other studies in an attempt to have a measurable way that the obstetricians can predict the outcome of the trial of labor in this group of women [28, 29]. However, the previous studies have not tested the ideal thickness of the LUS and myometrium in the active stage of labor in women with a previous one cesarean section, which is the main strength of our study.

The thickness of the LUS with cesarean section area changes throughout pregnancy. The area of the cesarean section scar is expanded while the fetus is growing. The wall of the uterine body as a whole and the scar area become thin simultaneously [28]. It was demonstrated that women with previous low transverse cesarean delivery have a LUS thickness at the term of pregnancy 0.9 mm thinner than women without previous cesarean delivery [30]. This will lead to the idea that LUS thickness in labor in this group of women is also less than the physiological one during labor. As suggested by several authors, the degree of LUS thinning, when measured by echography near term, may be related to the functional status of the scarred LUS and thus to the risk of uterine rupture [13, 29].

We used LUS thickness and MT to determine their correlations with the uterine defect. Many published articles have elucidated that the risk of a defective scar at delivery is renovated with the degree of thinning of MT or full LUS, respectively [31–33].

The argument for the relationship between maternal and perinatal factors and the success of vaginal birth after cesarean section is conflicting regarding the factors reported in published studies. Examples of these factors include age, maternal education level, gestational age, parity, number of abortions, obesity, diabetes, hypertensive disorders in pregnancy, Bishop score, labor induction, previous vaginal birth before cesarean section, previous vaginal birth after a cesarean section, and the indications for the previous cesarean section [34–36]. The current study assessed the effect of maternal age groups, gestational age, BMI, inter pregnancy and inter delivery interval, and birth weight. The findings also conflicted with the other published studies concerning maternal and perinatal characters and their effects on vaginal birth after a cesarean section.

On the other hand, our results did not show any significant relation between maternal characters and an increased rate of the uterine defect.

Uterine rupture is one of the most devastating complications of attempting a trial of labor after cesarean section, and the risk varies based on the location of the uterine incision. The risk of rupture amongst women with a previous low transverse uterine incision ranges from 0.7 to 0.9%. The current study reported uterine rupture in four women (2.5%). This very high rate emphasizes the need to appropriately decide on the mode of delivery for these women with a previous cesarean section and attempting vaginal delivery without previous counseling. It is crucial to make a timely decision whether to plan for an emergency cesarean section or continue giving the chance of vaginal delivery [24, 37, 38].

The liability of intensive labor supervision, including continuous intrapartum care to monitor progress in labor [39] and sonographic assessment of LUS thickness in labor, may provide more warranty and better perspicacity into uterine activity and thus a lower risk of uterine rupture [32].

To date, our study is considered the first study to be conducted on women with previous cesarean section and in the active first stage of labor in whom a sonographic assessment of the LUS thickness and MT was conducted in a very busy hospital. All women were followed up until the delivery was completed. Another strength of this study is that the reproducibility of these measurements as the LUS thickness and the MT were conducted by two observers and by two methods of ultrasound measurements, vaginal and abdominal. The reproducibility between two observers improves the reliability of the measurements.

The relatively low sensitivity and positive and negative predictive values of LUS and MT in the current study might limit the clinical usefulness of LUS and MT. Similarly, a previous meta-analysis had supported the use of antenatal LUS measurements in predicting a uterine defect during a trial of labor. However, it recommended assessing clinical applicability in prospective observational studies using a standardized method of measurement [21]. Recently, studies using randomized control trials have been conducted to assess the efficacy of ultrasound measurement of the LUS in reducing fetal and maternal adverse outcomes [40]. However, most of these studies have measured LUS at early pregnancy rather than during a trial of labor [13, 40].

This study has several limitations. It was not a randomized controlled trial. The data about the trial of labor and cesarean delivery were obtained from an observational study that lacks the comparability between women undergoing a labor trial and those undergoing elective repeat cesarean delivery. Not conducting the reproducibility of the ultrasound measurement is another limitation of this study. Although previous research has shown that the intra- and inter-observer differences were 1 mm or less, reproducibility of ultrasound measurement of the LUS and MT is still important, as the measurement in previous research was not during labor. The uterine contractions and the degree of cervical dilation can affect the measurements by LUS and MT. Therefore, the reliability of measured values should have been considered.

Having four cases of uterine rupture is also an important limitation of this study. Most women were interviewed for the first time during labor, having no previous antenatal examination to plan for the mode of delivery. This may increase the risk of the uterine defect. However, as we mentioned before, this problem is usually encountered in low-income countries and hospitals. This was one of our main inclusion criteria to predict uterine rupture before the too long trial of labor.

## Conclusion

In conclusion, a full LUS thickness of 2.3 mm and MT of 1.9 mm at 34–41 weeks of gestation in women during the active first stage of labor is associated with a high risk of complete uterine rupture during a trial of labor. Therefore, measurement of full LUS thickness and MT during labor can have a practical application in deciding the mode of delivery in women who had previously given birth by cesarean section and desired to have a vaginal birth and may lead to a reduction of uterine rupture.

## Data Availability

The datasets generated and/or analyzed during the current study are available in the Mendeley Datasets repository, https://doi.org/10.17632/2w95yvsg9s.1.

## Abbreviations

LUS: Lower Uterine Segment
MT: Myometrial Thickness
